# Sensitive detection of gallic acid in food by electrochemical sensor fabricated by integrating nanochannel film with nanocarbon nanocomposite

**DOI:** 10.3389/fnut.2024.1491345

**Published:** 2024-11-12

**Authors:** Jiasheng Li, Jiahui Lin, Tao Luo, Junjie Liu, Jiyang Liu, Wuning Zhong

**Affiliations:** ^1^Guangxi Medical University Cancer Hospital, Nanning, China; ^2^School of Chemistry and Chemical Engineering, Zhejiang Sci-Tech University, Hangzhou, China

**Keywords:** electrochemical sensor, vertically-ordered mesoporous silica film, electrochemical reduced graphene, graphene quantum dots, gallic acid

## Abstract

Sensitive detection of gallic acid (GA) in foods is of great significance for assessing the antioxidant properties of products and ensuring consumer health. In this work, a simple electrochemical sensor was conveniently fabricated by integrating vertically-ordered mesoporous silica film (VMSF) with electrochemically reduced graphene oxide (ErGO) and nitrogen graphene quantum dots (NGQDs) nanocomposite, enabling sensitive detection of GA in food sample. A water-soluble mixture of graphene oxide (GO) and NGQDs was drop-cast onto the common carbon electrode, glassy carbon electrode (GCE), followed by rapid growth of VMSF using an electrochemically assisted self-assembly method (EASA). The negative voltage applied during VMSF growth facilitated the *in situ* reduction of GO to ErGO. The synergistic effects of ErGO, NGQDs, and the nanochannels of VMSF led to nearly a tenfold enhancement of the GA signal compared to that obtained on electrodes modified with either ErGO or NGQDs alone. Sensitive detection of GA was realized with a linear concentration range from 0.1 to 10 μM, and from 10 to 100 μM. The limit of detection (LOD), determined based on a signal-to-noise ratio of three (*S*/*N* = 3), was found to be 81 nM. Combined with the size-exclusion property of VMSF, the fabricated sensor demonstrated high selectivity, making it suitable for the sensitive electrochemical detection of gallic acid in food samples.

## 1 Introduction

Gallic acid (GA) is a natural polyphenolic compound commonly found in plants, particularly in tea leaves, grapes, oak bark, and certain fruits (1). Due to its potent antioxidant properties, GA is widely used in various fields, including food, medicine, and cosmetics (2). As a powerful antioxidant, GA can delay the oxidation process in foods, thereby extending their shelf life. It also neutralizes free radicals in the body, slows cellular aging, and reduces the risk of chronic diseases (3). Beyond its antioxidant effects, GA also exhibits anti-inflammatory, antimicrobial, and anticancer properties, contributing to the prevention and treatment of related diseases (4–6). In cosmetics, GA helps prevent skin oxidation, delay aging, and maintain skin health. However, excessive intake of gallic acid can pose health risks. It has moderate toxicity, with a probable oral lethal dose for humans ranging from 0.5 to 5 g/kg (7). High doses may burden the liver and kidneys, potentially causing damage to these organs, and can lead to gastrointestinal discomfort (e.g., nausea and diarrhea). Additionally, GA may inhibit iron absorption, leading to iron deficiency anemia, which poses particular risks to pregnant women and children (8). Therefore, sensitive detection of GA in foods is crucial for evaluating the antioxidant properties of products and ensuring consumer health.

Currently, the methods for GA detection in real food samples primarily include high-performance liquid chromatography (HPLC), capillary electrophoresis (CE), and electrochemical detection (9–14). HPLC offers high sensitivity and excellent separation capabilities, enabling accurate determination of GA and effective separation from interfering substances. However, it suffers from high equipment costs and complex operation. CE provides high separation efficiency, rapid analysis, and low sample consumption, but demands a high level of technical expertise. Electrochemical detection is advantageous due to its rapid detection, simple instrumentation, and the ease of electrode integration and miniaturization (15–18). By modifying electrodes to enhance analyte enrichment, detection sensitivity can be significantly improved. However, electrodes are prone to contamination, leading to poor reproducibility (19). Developing electrochemical sensors with high sensitivity and strong anti-interference capability for GA detection in food is highly desirable.

Incorporating nanomaterials onto electrodes to enrich analytes is an effective approach for enhancing the sensitivity of electrochemical sensors (20–23). Among these materials, carbon-based nanomaterials have garnered significant attention due to their unique physical and chemical properties (24–26). For example, graphene materials offer advantages such as high electrical conductivity, large surface area, and ease of functionalization (27–29). When used to modify electrodes, their excellent electron mobility can enhance the efficiency of electrochemical reactions, while their two-dimensional (2D) structure provides large surface area, increasing the number of active sites on the electrode surface (30). However, graphene tends to aggregate, leading to poor dispersion in solutions, which can negatively impact the uniformity and performance of the electrode modification. Compared to chemical vapor deposition (CVD) methods for producing graphene, using highly water-soluble graphene oxide (GO) to modify electrodes, followed by electrochemical reduction to obtain reduced graphene oxide (ErGO), is a more convenient approach. ErGO retains part of *π–*π conjugated structure of graphene, ensuring good electrical conductivity, while also maintaining a certain number of oxygen-containing functional groups on its surface (e.g., hydroxyl, carboxyl, and epoxy groups). These functional groups can serve as active sites that interact with analytes, providing electrocatalytic properties (31). Additionally, zero-dimensional (0D) graphene, particularly graphene quantum dots (GQDs), has attracted significant attentions as a novel carbon nanomaterial (32–36). The nanoscale size and 0D structure of GQDs result in a large specific surface area, offering more active sites and high catalytic activity (37–41). However, due to their extremely small size, GQDs may face challenges such as detachment or aggregation during usage, potentially affecting the long-term stability of the modified electrodes. By combining graphene with GQDs to fabricate composite nanomaterials modified electrodes, the advantages of both materials can be synergistically utilized. On the one hand, graphene provides a large 2D surface, while GQDs contribute additional nanoscale active sites. The composite structure can significantly increase the active surface area, thereby enhancing enrichment and catalytic performance. On the other hand, the high mechanical strength of graphene reinforces the structural stability and durability of the modified electrode. In addition, the composite materials can overcome the drawbacks of graphene’s tendency to agglomerate and the low conductivity of GQDs. Thus, simple fabrication of graphene and GQDs nanocomposite modified electrodes holds great potential in electrochemical detection with high sensitivity.

Enhancing resistance of the electrode to interference from complex matrices is also crucial in electrochemical sensors. Common strategies include surface modification and the use of selective films (42–44). Surface modification involves introducing functional materials, such as molecularly imprinted polymers or biomolecules, onto the electrode surface, allowing for selectively adsorb target substances and reduce interference from other components in the matrix (45–48). However, these modification materials can swell during use, leading to instability of the electrode. Covering the electrode surface with a selective permeable film that allows only target molecules to pass through while blocking interfering substances can significantly enhance the anti-interference capability of electrode in real sample analysis. Recent studies have demonstrated that introducing vertically-ordered mesoporous silica film (VMSF) on the electrode surface is an effective strategy for improving electrode performance for highly selective detection in complex matrices (49–53). VMSF features ultra-small pore sizes (typically 2–3 nm), allowing selective diffusion of small molecules while blocking larger molecules or interfering substances. For instance, the size-exclusion effect prevents large molecules such as proteins, organelles, and suspended particles from entering the mesopores (54–56). The insulating nature of silica structure of VMSF helps prevent contamination of the electrode by the corresponding non-specific adsorption, significant enhancing the anti-fouling ability of electrode (57). Moreover, the low p*K*_a_ value (~2) of silanol groups in VMSF results in a negatively charged surface in conventional solutions, which effectively reduces the electrochemical signals of negatively charged interfering substances in the matrix, such as uric acid (UA) and ascorbic acid (AA), thereby reducing interference (58, 59). Silica materials also exhibit excellent chemical stability and mechanical strength, preventing swelling during long-term use and improving electrode stability.

In this work, an electrochemical sensor was easily fabricated by integrating VMSF with electrochemically reduced graphene oxide (ErGO) and nitrogen graphene quantum dots (NGQDs) nanocomposite, which can realize highly sensitive detection of GA in food samples. A water-soluble mixture of GO and GQDs was drop-cast onto the common carbon electrode, glassy carbon electrode (GCE), followed by rapid growth of VMSF using an electrochemically assisted self-assembly method (EASA). The negative voltage applied during VMSF preparation facilitated the *in situ* reduction of GO to ErGO. The synergistic effects of ErGO, NGQDs, and the nanochannels of VMSF enhanced GA signal. Coupled with the size-exclusion property of VMSF, this sensor exhibited high anti-interference capabilities, making it suitable for sensitive electrochemical detection of GA in food samples.

## 2 Materials and methods

### 2.1 Chemicals and materials

Tetraethoxysiloxane (TEOS), cetyltrimethylammonium bromide (CTAB), potassium ferricyanide (K_3_[Fe(CN)_6_]), potassium ferrocyanide (K_4_[Fe(CN)_6_]), sodium dihydrogen phosphate dihydrate (NaH_2_PO_4_·2H_2_O), disodium hydrogen phosphate dodecahydrate (Na_2_HPO_4_·12H_2_O), anhydrous sodium acetate, acetic acid, gallic acid (GA), glucose (Glu), leucine (Leu), and arginine (Arg) were all purchased from Aladdin Biochemical Technology Co., Ltd. (Shanghai, China). Ethanol (99.8%) and concentrated hydrochloric acid (HCl, 38%) were obtained from Shuanglin Chemical Reagent Co., Ltd. (Hangzhou, China). Sodium chloride (NaCl), sodium nitrate (NaNO_3_), and potassium chloride (KCl) were sourced from Gaojing Chemicals Co., Ltd. (Hangzhou, China). Green tea was purchased from a local supermarket (Hangzhou, China). Phosphate-buffered saline (PBS) was prepared from Na_2_HPO_4_ and NaH_2_PO_4_, and acetate buffer solution (ABS) was prepared from sodium acetate and acetic acid. All chemicals and reagents were of analytical grade and used without further purification. The aqueous solutions used in the experiments were prepared with ultrapure water (18.2 MΩ•cm).

### 2.2 Characterization and instrumentations

The morphology and thickness of VMSF were characterized using transmission electron microscopy (TEM, HT7700, Hitachi, Japan). The preparation of TEM samples involved two steps. Firstly, VMSF was gently scraped off the electrode surface with a blade and dispersed in ethanol under ultrasonic treatment for 40 min. The resulting dispersion was then drop-cast onto a copper grid and dried under an infrared lamp before TEM analysis. For NGQDs characterization, TEM sample was prepared on an ultrathin carbon film. The sample was prepared using the dip-coating method using NGQDs solution (0.5 mg/mL) and then dried. UV–vis spectra were obtained using a UV–Vis spectrophotometer (UV-2450, Shimadzu, Japan). Electrochemical experiments, including cyclic voltammetry (CV) and differential pulse voltammetry (DPV), were performed on an Autolab electrochemical workstation (PGSTAT302N, Switzerland). All electrochemical experiments were conducted using the traditional three-electrode system, with an Ag/AgCl electrode as the reference electrode, a platinum wire or plate as the counter electrode, and a bare or modified GCE as the working electrode. The DPV parameters were set as follows: pulse amplitude of 25 mV, step potential of 5 mV, pulse time of 0.05 s, and interval time of 0.2 s.

### 2.3 Synthesis of NGQDs

The NGQDs were synthesized using 1-aminopyrene as the carbon precursor under alkaline conditions through a hydrothermal method. Specifically, 1-aminopyrene (2 mg/mL) was added to 20 mL of ammonia solution (0.4 M). The mixture was then placed in a Teflon-lined autoclave for high-pressure reaction at 200°C for 10 h. After the reaction, the resulting solution was filtered through a 0.22 μm membrane to remove large particles, yielding a reddish-brown solution. The solution was then dialyzed for 2 days using dialysis bags with molecular weight cut-off of 500 Da to remove unreacted small molecules. Finally, the dialysate was freeze-dried to obtain solid NGQDs.

### 2.4 Fabrication of VMSF/NGQDs-ErGO/GCE

First, GCE was cleaned. Specifically, GCE (diameter = 3 mm) was sequentially polished with alumina powders of 0.5, 0.3, and 0.05 μm, followed by ultrasonic cleaning in ethanol and ultrapure water. Then, mixture containing NGQD (0.03 M) and GO (0.1 M) was prepared 1.0 mL. The NGQDs-GO solution (1.0 mL) was then drop-cast onto the surface of cleaned GCE and dried at 60°C for 20 min to obtain the modified electrode, labeled as NGQDs-GO/GCE. Subsequently, the electrochemical-assisted self-assembly (EASA) method was used to rapidly grow VMSF on the NGQDs-GO/GCE (60). During the growth of VMSF, GO is *in situ* electrochemically reduced to ErGO. The precursor solution for VMSF growth was prepared by adding CTAB (1.585 g) and TEOS (3,050 mL) in the solution containing ethanol (20 mL) and sodium nitrate (NaNO_3_) and stirring at room temperature for 2.5 h. The three-electrode system was immersed in the precursor solution with NGQDs-GO/GCE as the working electrode. A constant voltage of-2.2 V was applied for 5 s. Then, the electrode was quickly removed, thoroughly washed with ultrapure water, and aged overnight at 80°C. The resulting electrode contains CTAB micelles (SM) within the nanochannels was labeled as SM@VMSF/NGQDs-ErGO/GCE. Finally, the SM@VMSF/NGQDs-ErGO/GCE was placed in HCl (0.1 M) in ethanol medium and stirred for 5 min to remove the SM, resulting in the open nanochannel modified electrode, denoted as VMSF/NGQDs-ErGO/GCE.

### 2.5 Electrochemical detection of GA using VMSF/NGQDs-ErGO/GCE

When detecting GA with VMSF/NGQDs-ErGO/GCE, a supporting electrolyte solution of ABS (0.1 M, pH = 2) was used. The DPV response of different concentrations of GA was measured. For real sample analysis, the GA content in green tea was determined. Specifically, 0.20 g of green tea was added to the extraction solution containing ultrapure water (15 mL) and ethanol (35 mL) as the solvent. After ultrasonic extraction for 2 h, the sample was centrifuged at 5000 rpm for 30 min. Then, the supernatant was collected for determination.

## 3 Results and discussion

### 3.1 Fabricating electrochemical sensor by integrating with VMSF using *in situ* prepared NGQDs-ErGO as conductive adhesive layer

**Figure 1:**
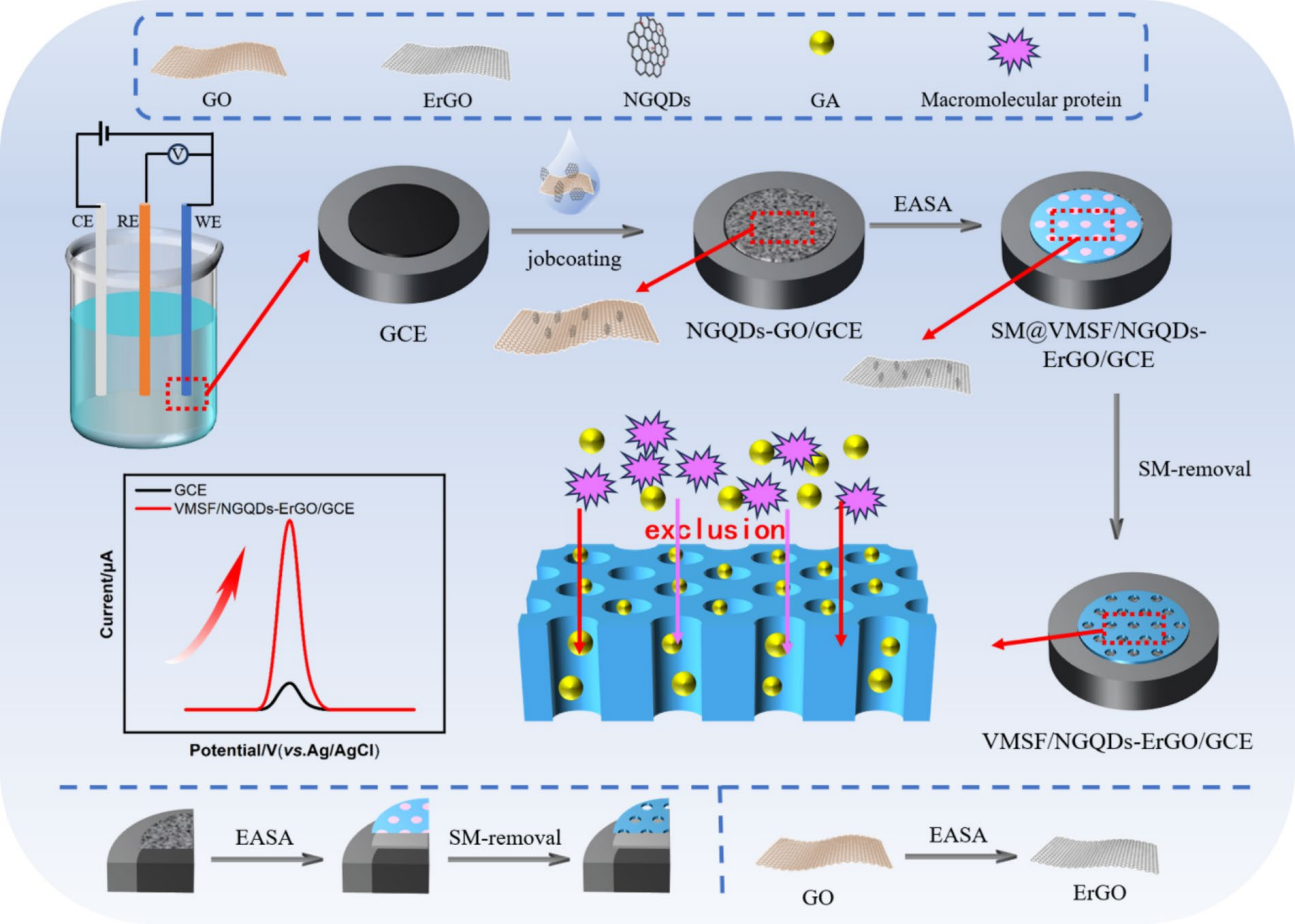
Illustration for the fabrication of VMSF/NGQDs-ErGO/GCE sensor for electrochemical detection of GA by integrating VMSF with *in situ* formed ErGO and NGQDs nanocomposite.

In this work, a simple electrochemical sensor was efficiently developed by employing nitrogen-doped graphene quantum dots (NGQDs) combined with electrochemically reduced graphene oxide (ErGO) as a conductive layer. As illustrated in Figure 1, this approach integrated glassy carbon electrode (GCE) with an anti-fouling layer of vertically-ordered mesoporous silica film (VMSF) to construct a high-performance sensor. Initially, graphene oxide (GO) was modified onto GCE, followed by the growth of VMSF was grown using the electro-assisted self-assembly (EASA) method, which simultaneously reduced GO to ErGO in one step. The EASA method employs a surfactant as a template to control the interfacial self-assembly of surfactants and the sol–gel reaction of siloxane precursors on the electrode, facilitating the stable growth of VMSF on the electrode surface. Specifically, by applying a negative potential on the electrode, water or hydrogen ions were reduced, creating an *in situ* pH gradient on the electrode surface. This induced the self-assembly of CTAB micelles on the electrode surface and catalyzed the hydrolysis and polycondensation of siloxane precursor. As a result, vertically-aligned and nanometer-sized channels filled with surfactant micelles (SM) are formed on the electrode surface. After removing SM with a hydrochloric acid-ethanol solution, open nanochannels were obtained on the modified electrode surface. The *π*-conjugated hydrophobic structure, 2D planar architecture, and surface oxygen-containing groups of NGQDs and ErGO facilitated the stable growth of VMSF. For instance, the abundant oxygen-containing groups (e.g., -OH) on NGQDs and ErGO can undergo co-condensation reactions with silanol groups, forming O-Si-O bonds and enhancing the adhesion stability of VMSF. Simultaneously, the growth of VMSF also improved the stability of NGQDs and ErGO on GCE. The high electron transfer rate of ErGO, combined with the synergistic enrichment of gallic acid (GA) by NGQDs, ErGO, and VMSF, significantly enhanced the detection performance of the fabricated electrochemical sensor.

**Figure 2:**
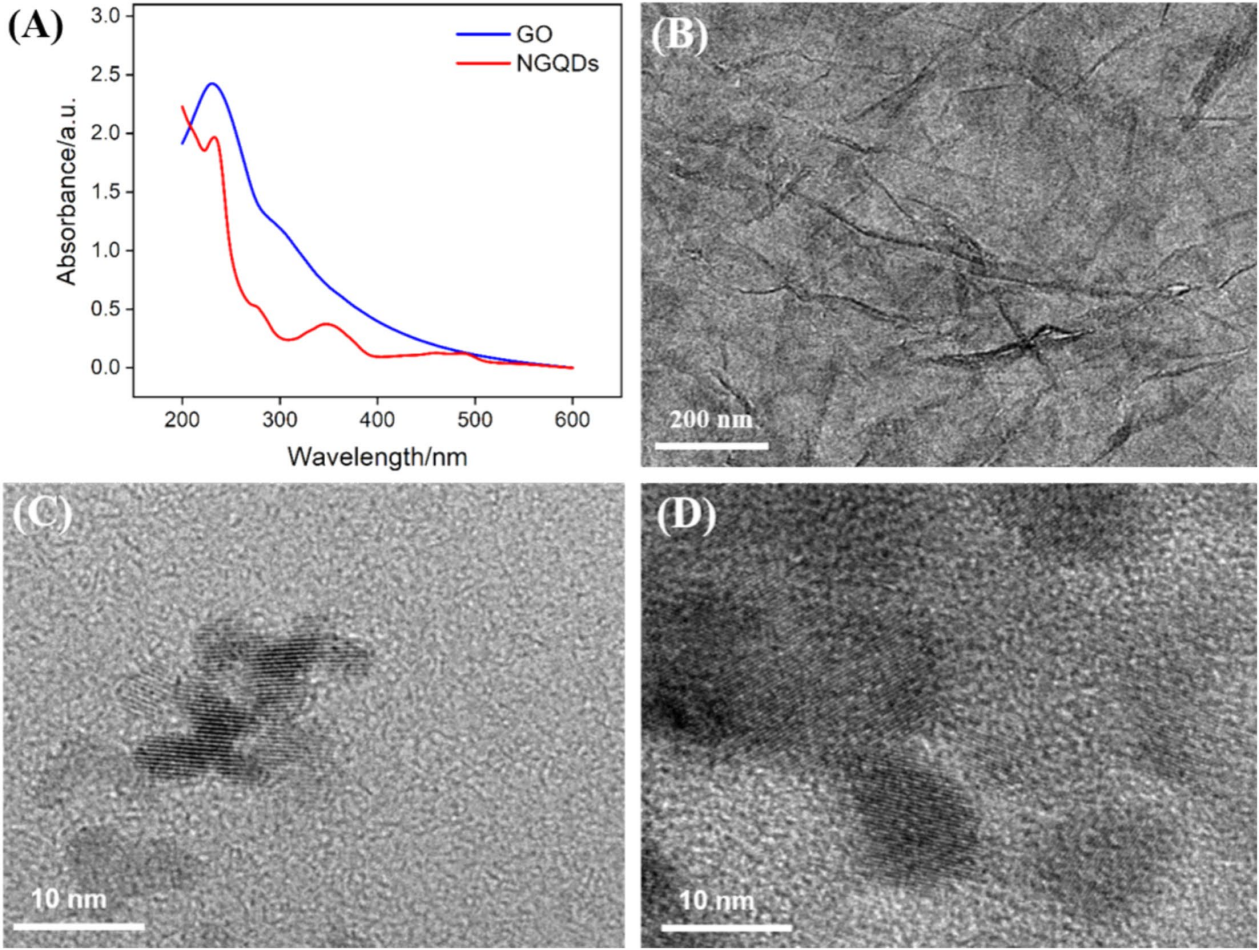
(A) UV–Vis absorption spectra of NGQDs (0.3 mg/mL) and GO (0.1 mg/mL). (B–D) TEM image of GO (B), NGQDs (C), and NGQDs-GO nanocomposite (D) at high resolution.

The NGQDs were synthesized using a one-step hydrothermal method using 1-aminopyrene as the carbon precursor and ammonia as the nitrogen source. As shown in Figure 2A, NGQDs exhibited a distinct absorption peak at 230 nm, corresponding to the conjugated structure of sp^2^ carbon in their graphene core. A significant UV absorption feature appeared around 350 nm, attributed to the n → π* transition of oxygen-containing groups such as C=O. The UV absorption spectrum of GO also had an absorption peak at 230 nm, associated with the conjugated structure of sp^2^ carbon, along with a shoulder peak around 300 nm, which was related to the n → π* transition of its surface functional groups.

The morphology of GO, NGQDs, and the NGQDs-ErGO composite was characterized using TEM. Figure 2B displayed the TEM image of GO, revealing a monolayer sheet structure with noticeable wrinkles. Figure 2C displayed the high-resolution TEM (HRTEM) image of NGQDs, where clear lattice fringes were observed with a lattice spacing of 0.35 nm, consistent with the (100) crystal plane of graphene. After the growth of VMSF, the VMSF/ErGO/GCE was treated with 1 M NaOH solution to remove VMSF, yielding the NGQDs-ErGO substrate. Figure 2D exhibited the HRTEM image of NGQDs-GO, where the lattice structure of NGQDs distributed on ErGO was visible.

**Figure 3:**
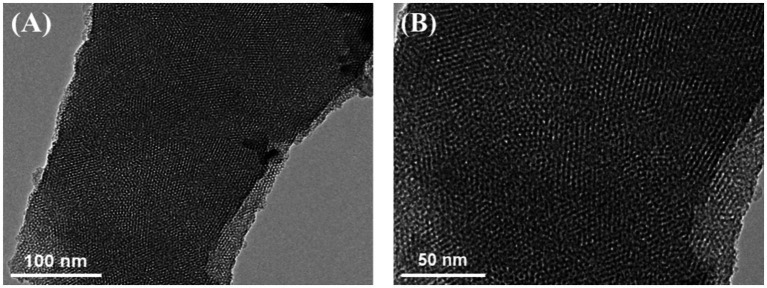
(A) Top-view TEM image of VMSF. (B) Top-view HRTEM image of VMSF.

The microstructure and morphology of VMSF were characterized using TEM. Figure 3A showed a top-view TEM image of VMSF, where the surface appears intact, without cracks or significant defects over a large area. In the HRTEM image (Figure 3B), the surface displayed a well-ordered hexagonal arrangement of nanochannels with uniform size, and the pore diameter was approximately 2.2 nm.

### 3.2 Characterization of electrode modification process and the enhanced electrochemical signal of GA on VMSF/NGQDs-ErGO/GCE

**Figure 4:**
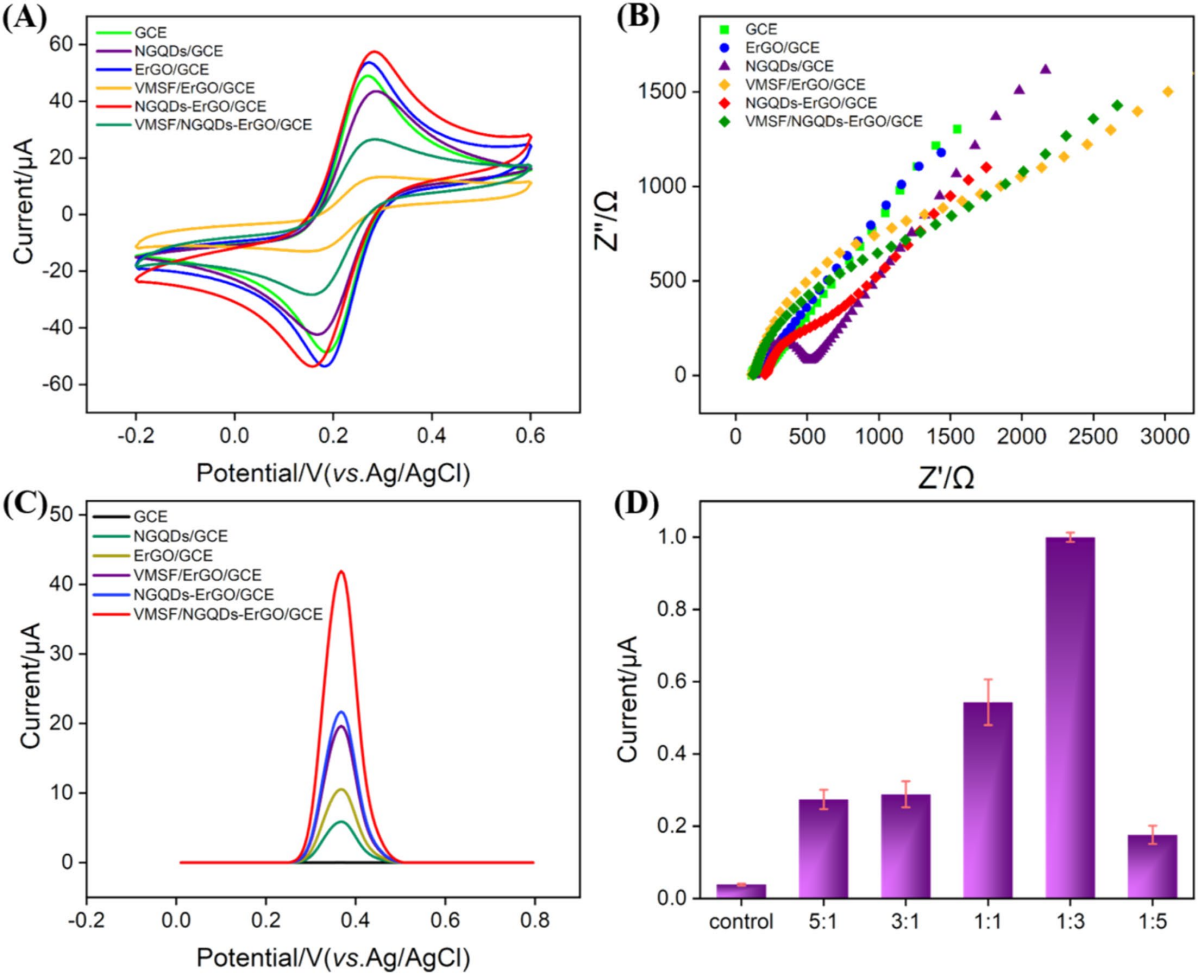
Cyclic voltammetry (CV) (A) and Nyquist plots (B) obtained on different electrodes in 0.1 M KCl solution containing 2.5 mM [Fe(CN)_6_]^3−/4−^. (C) The DPV curves obtained on different electrodes in GA solution. (D) The DPV currents obtained on VMSF/NGQDs-ErGO/GCE fabricated using different ratio of NGQDs and GO.

To investigate the successful modification of the electrode and the impact of various nanomaterials on sensor performance, electrodes modified NGQDs, ErGO, VMSF/ErGO, and NGQDs-ErGO were fabricated. As shown in Figure 4A, the cyclic voltammetry (CV) signals of different electrodes were compared with those of bare GCE and VMSF/NGQDs-ErGO/GCEs using the standard redox probe Fe(CN)_6_^3−/4−^. When NGQDs were modified on the electrode surface, the redox peak current of Fe(CN)_6_^3−/4−^ decreased, and the peak-to-peak separation increased. This phenomenon was attributed to the high oxygen-containing edge groups of NGQDs, which reduced electron transfer and the reversibility of the redox reaction on the electrode. When ErGO was modified on the electrode surface, the measured peak current was higher than that of the GCE, attributed to the high electron transfer rate of ErGO. The peak current increased when NGQDs-ErGO was modified on the electrode surface, possibly due to the increased electrochemical surface area. When VMSF was modified on the electrode surface, the signals for VMSF/ErGO/GCE and VMSF/NGQDs-ErGO/GCE were significantly lower than that on GCE, due to the electrostatic repulsion of the negatively charged VMSF surface against the negatively charged Fe(CN)_6_^3−/4−^. Figure 4B was the EIS curves of different electrodes. The charge transfer resistance (*R*et) of GCE was measured to be 372 Ω, while the *R*et values for NGQDs, ErGO, and NGQDs-ErGO modified GCE were 563 Ω, 59 Ω, and 295 Ω, respectively. The modification of GCE with NGQDs led to increased impedance of the modified electrode. Due to the good conductivity of ErGO, the *R*et of the ErGO/GCE decreased. When both NGQDs and ErGO were present, the *R*et of the electrode increased compared to ErGO/GCE. The *R*et values for VMSF/NGQDs-ErGO/GCE and VMSF/ErGO/GCEs with VMSF modification were 687 and 585 Ω, respectively. The negatively charged surface of VMSF repelled the negatively charged probe, increasing the interfacial resistance of the electrode. These results confirmed the successful construction of the sensor.

The current signals of GA on different modified electrodes were measured and compared using DPV. As shown in Figure 4C, the DPV oxidation peak current signals increased for NGQDs, ErGO, and VMSF/NGQDs-ErGO modified electrodes compared to bare GCE. This enhancement was attributed to the increased conductivity of ErGO, as well as the enrichment effects of NGQDs, ErGO, and VMSF on the analyte. The synergistic effect of NGQDs-ErGO and VMSF resulted in nearly a 10-fold increase in the signal intensity for GA on the VMSF/NGQDs-ErGO/GCE, which is advantageous for high-sensitivity detection of GA. The signals of GA on VMSF/NGQDs-ErGO/GCEs varied with different ratios of mixed NGQDs and GO. As shown in Figure 4D, the oxidation peak current signal of GA on the electrode first increased and then decreased as the GO ratio decreased. The peak current was highest when the GO ratio was 1:3.

### 3.3 Optimization of detection conditions

**Figure 5:**
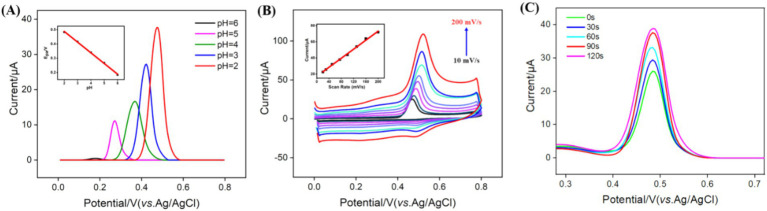
(A) DPV curves obtained on VMSF/NGQDs-ErGO/GCE in GA solution at different pH. The inset shows the relationship between peak potential and pH. Inset is the linear relationship between the peak current and pH. (B) CV curves obtained on VMSF/NGQDs-ErGO/GCE at different scan rate. Inset shows the linear relationship between the oxidation potential and scan rate. (C) DPV curves obtained at different enrichment times.

To enhance detection sensitivity, the pH for GA detection was optimized. As shown in Figure 5A, the highest DPV peak current signal for GA was observed at pH 2 on the VMSF/NGQDs-ErGO/GCE. This enhancement may be attributed to the influence of pH on the interaction between GA and the nanomaterials. At higher pH levels, GA carried a negative charge (p*K*_a_ ~ 4) and experienced electrostatic repulsion from the negatively charged VMSF, leading to a decrease in peak current. Additionally, it was found that as the pH increased, the DPV oxidation peak potential of GA shifted negatively, indicating that the electrode reaction involves proton participation. A linear regression of the oxidation peak potential (*E*_pa_) versus pH yielded a slope of −0.0751, which was close to the theoretical value of −0.0591 in Nernst equation, suggesting that the number of electrons and protons transferred in the electrooxidation of GA is equivalent.

To further explore the electrochemical behavior of GA on VMSF/NGQDs-ErGO/GCE, the effect of scan rate on the GA redox process was examined. Figure 5B displayed the CV curves of GA on VMSF/NGQDs-ErGO/GCE at varying scan rates. As observed, the peak current of GA increased with the scan rate, demonstrating a good linear relationship between the current and the scan rate (*v*) (inset of Figure 5B). This indicated that the electrochemical reaction of GA on electrode was adsorption-controlled. The enrichment time for GA detection was optimized and results were displayed in Figure 5C. As shown, the current intensity for GA increased with enrichment time, reaching equilibrium at 90 s. This may be attributed to the fact that, as the enrichment time increases, the adsorption sites in VMSF became saturated with GA, resulting in no further increase in the GA signal with additional enrichment time. Thus, 90 s was chosen as the optimal enrichment time.

### 3.4 Electrochemical detection of GA

**Figure 6:**
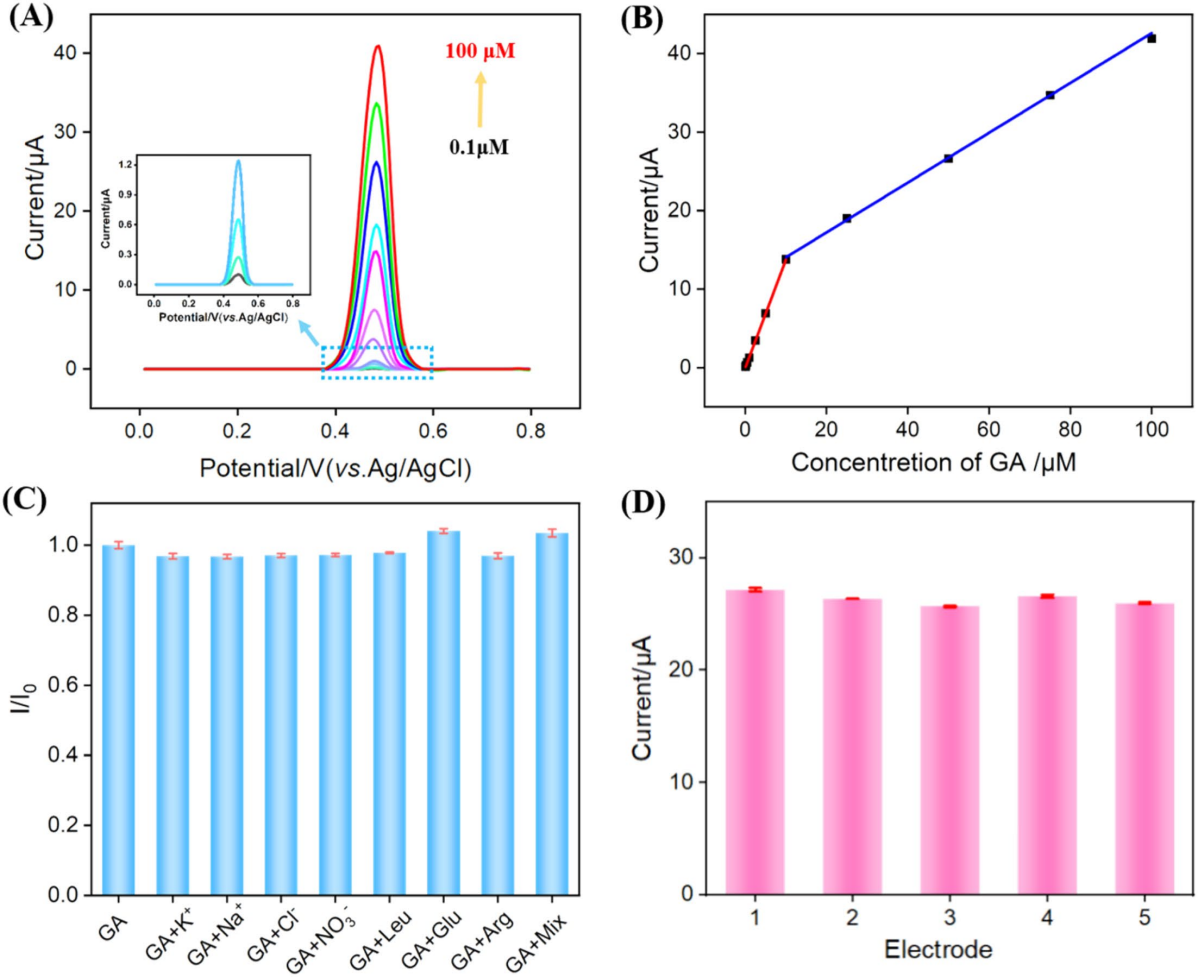
(A) DPV response to different GA concentrations. (B) The linear relationship between *I*_pa_ and GA concentration. (C) The rate of change of DPV signal in the absence (_I0_) and presence (*I*) of different substance or their mixture. (D) DPV response obtained on five independent electrodes for GA detection (50 μM).

Under the optimal detection conditions, a series of GA solutions with varying concentrations were analyzed using VMSF/NGQDs-ErGO/GCE, as shown in Figure 6A. As the GA concentration increased, the peak current continually increased. The oxidation peak current (*I*pa) of GA was linearly fitted with GA concentration (*C*_GA_). For GA concentrations ranging from 0.1 μM to 10 μM, the linear regression equation is *I*_pa_ = 1.38 *C*_GA_ – 0.0281 (*R*^2^ = 0.998). For GA concentrations ranging from 10 to 100 μM, the linear regression equation is *I*_pa_ = 0.318*C*_GA_ – 10.9 (*R*^2^ = 0.998, Figure 6B). The limit of detection (LOD) for VMSF/NGQDs-ErGO/GCE was calculated to be 81 nM, based on a signal-to-noise ratio of three (*S*/*N* = 3). Comparison of GA detection performance using different methods was displayed in Supplementary Table S1 in supporting information [SI, (61–66)]. The LOD obtained on the fabricated sensor was lower than that obtained using DPV detection by polyquercetin and multi-walled carbon nanotubes [Polyquercetin/MWCNT, (61)] or poly (pyrocatechol violet) and polyaminobenzene sulfonic acid functionalized single-walled carbon nanotubes [polyPCV/f-SWNT, (66)], or square wave voltammetry (SWV) detection by reduced graphene oxide [rGO, (63)], or CV detection by bismuth-nanoparticles decorated multi-walled carbon nanotubes cast-coated modified carbon paste electrode [Bi-MWCNT/MCPE, (64)], or amperometry (AMP) detection by silver nanoparticle and delphinidin [AgNPs/Delph, (65)]. The started concentration in linear detection range is lower than that obtained using electrochemiluminescence (ECL) detection by g-C_3_N_4_ and multi-walled carbon nanotubes composite material [g-C_3_N_4_@MWCNT, (62)].

### 3.5 Selectivity and reproducibility of the sensor

The selectivity and reproducibility of the prepared sensor were evaluated. The electrochemical signals were measured on VMSF/NGQDs-ErGO/GCE for GA alone or GA and one of common substances in food, including ions (K^+^, Na^+^, Cl^−^, NO₃^−^), glucose (Glu), leucine (Leu), and arginine (Arg), and their mixture (mix). As shown in Figure 6C, even when the concentrations of interfering substances were 10 times higher than that of GA, the electrochemical signal on the electrode was nearly identical to that observed for GA alone. On one hand, the modification of electrode with VMSF can reduce non-specific adsorption on the electrode surface, leading to stable signal (67–69). On the other hand, due to the electrode’s potential differentiation capability, the substances do not produce electrochemical signals on the electrode within the measured electrochemical window. Figure 6D showed that the relative standard deviation (RSD) of the DPV signals for GA measured with five VMSF/NGQDs-ErGO/GCEs was 2.2%, demonstrating good reproducibility of the sensor.

### 3.6 Real sample analysis

To further investigate the detection performance of VMSF/NGQDs-ErGO/GCE for GA in real samples, the standard addition method was employed to determine the GA content in green tea. The sample required no chemical pretreatment and was directly added to the test electrolyte solution for electrochemical detection. The concentration of GA in actual samples can be derived from the linear extrapolation from the standard addition method, which involves determining the intersection point of the extended linear curve of the electrochemical signal versus GA concentration with the x-axis. Specifically, this point indicates the concentration of GA in the real sample. After adding a solution of known concentration to the sample, the corresponding recovery rate and the RSD of the signal can be measured to evaluate the accuracy of the detection method. Generally, a recovery rate between 90 and 110% and an RSD below 5% indicate high detection accuracy. As shown in Table 1, the GA content in the green tea sample was measured to be 84 μM. Additionally, the VMSF/NGQDs-ErGO/GCE exhibited satisfactory recovery rates (100 ~ 106%) and low RSD values (<3.5%), demonstrating good accuracy of the detection.

**Table 1:** Detection of GA in green tea by VMSF/NGQDs-ErGO/GCE sensor.

Sample	Added (μM)	Found (μM)	RSD (%, *n* = 3)	Recovery (%)
Green tea	0.00	8.40	–	–
	1.00	9.41	3.5	100
	20.0	29.8	3.0	106

## 4 Conclusion

In summary, VMSF modified carbon-based electrode was fabricated by using *in situ* formed ErGO and NGQDs nanocomposite as conductive layer. VMSF was directly grown via a rapid EASA method, accompanying with *in situ* formation of ErGO from GO, ensuring the stable integration of VMSF. The fabrication of the sensor is simple. The synergistic effects of ErGO, NGQDs, and the nanochannels of VMSF result in significantly enhanced GA signal in comparison with that obtained on ErGO or NGQDs modified electrode. The sensor exhibits wide linear relationship ranged from 0.1 to 10 μM and from 10 to 100 μM with low detection limit of 0.081 μM. The fabricated sensor had high selectivity and good reproducibility. Determination of GA content in green tea was realized. In this work, although glassy carbon electrode is the most common type of carbon electrode, its relatively high cost may limit the large-scale production or use of the constructed sensors. Future studies could combine this method with screen-printed electrodes or patterned indium tin oxide (ITO) to develop disposable or single-use sensors. This easy method for fabrication of the electrochemical sensor coupled with the high detection performance, holds promising applications in direct analysis of GA in food samples.

## Figures and Tables

**Table:** 

GA	gallic acid
VMSF	vertically-ordered mesoporous silica film
ErGO	reduced graphene oxide
NGQDs	nitrogen graphene quantum dots
GO	graphene oxide
GCE	glassy carbon electrode
LOD	limit of detection
HPLC	high-performance liquid chromatography
UV–Vis	ultraviolet–visible spectroscopy
CE	capillary electrophoresis
2D	two-dimensional
CVD	chemical vapor deposition
0D	zero-dimensional
EASA	electrochemically assisted self-assembly method
TEOS	tetraethoxysiloxane
CTAB	cetyltrimethylammonium bromide
Glu	glucose
Leu	leucine
Arg	arginine
PBS	phosphate-buffered saline
CV	cyclic voltammetry
DPV	differential pulse voltammetry
LOD	limit of detection
MWCNT	multi-walled carbon nanotubes
f-SWNT	sulfonic acid functionalized single-walled carbon nanotubes
polyPCV	poly (pyrocatechol violet)
rGO	reduced graphene oxide
Bi-MWCNT/MCPE	bismuth-nanoparticles decorated multi-walled carbon-nanotubes cast-coated modified carbon paste electrode
AMP	amperometry
AgNPs	silver nanoparticle
ECL	electrochemiluminescence

## Data Availability

The original contributions presented in the study are included in the article/Supplementary material, further inquiries can be directed to the corresponding authors.
