# Supplementary material for: Sensitive detection of gallic acid in food by electrochemical sensor fabricated by integrating nanochannel film with nanocarbon nanocomposite
Source: Front Nutr. 2024 Nov 12;11:1491345. doi: 10.3389/fnut.2024.1491345 (PMC11588441; doi:10.3389/fnut.2024.1491345)
Supplement: Supplementary file 1 [file Table_1.DOCX]

Table S1 Comparison of GA detection performance using different methods.

| **Electrode materials** | **Methods** | **Detection range**  **(μM)** | **LOD**  **(μM)** | *Ref.* |
| --- | --- | --- | --- | --- |
| *polyquercetin/MWCNT/GCE* | DPV | 10-750 | 0.10 | 64 |
| *g-C_3_N_4_@MWCNT/GCE* | ECL | 0.01-10 | 0.002 | 65 |
| *rGO/GCE* | SWV | 8-400 | 0.42 | 66 |
| *Bi-MWCNT/MCPE* | CV | 1-100 | 0.16 | 67 |
| *AgNPs/Delph/GCE* | AMP | 8.68-625.8 | 0.28 | 68 |
| *polyPCV/f-SWNT/*  *GCE* | DPV | 0.75–10 and  10–100 | 0.12 | 69 |
| *VMSF/NGQD-ErGO/GCE* | DPV | 0.1-10 and  10-100 | 0.081 | This work |

MWCNT, multi-walled carbon nanotubes; GCE, glassy carbon electrode; ECL, electrochemiluminescence; rGO, reduced graphene oxide; SWV, square wave voltammetry; Bi-MWCNT, bismuth-nanoparticles decorated MWCNT; MCPE, cast-coated modiﬁed carbon paste electrode; AgNPs, silver nanoparticle; Delph, delphinidin; AMP, amperometry; polyPCV/f-SWNT, poly(pyrocatechol violet) and polyaminobenzene sulfonic acid functionalized single-walled carbon nanotubes.
